# Vertical Transmission of Herpes Simplex Virus Acquired After Primary Outbreak in Second Trimester of a Dichorionic Twin Gestation

**DOI:** 10.1155/S1064744997000690

**Published:** 1997

**Authors:** G. M. Joffe, K. Olson, T. L. Merlin, A. Brown

**Affiliations:** 1 Lovelace Medical Center Albuquerque NM USA; 2 Department of OB-GYN 5400 Gibson Blvd., S.E., Albuquerque NM 87108 USA

## Abstract

*Background:* The incidence of genital herpes simplex virus (HSV) has increased in recent years, particularly among women of reproductive age. This places more neonates at risk for severe morbidity and mortality. Treatment recommendations for primary disease in pregnancy are lacking, particularly for those who acquire. HSV remote from term.

*Case:* A patient at 17 weeks of gestation carrying dichorionic twins developed primary herpes with subsequent vertical transmission of the virus and significant neonatal morbidity.

*Conclusion:* Data regarding risks and benefits of treatments such as acyclovir and immunoprophylaxis are lacking at a time when the incidence of HSV infection is on the rise.


Infectious Diseases in Obstetrics and Gynecology 5:380–385 (1997)
© 1998 Wiley-Liss, Inc.

### Vertical Transmission of Herpes Simplex Virus Acquired After Primary Outbreak in Second Trimester of a Dichorionic Twin Gestation

G.M. Joffe,* K. Olson, T.L. Merlin, and A. Brown
*Lovelace Medical Center, Albuquerque, NM*

ABSTRACT

*Background:* The incidence of genital herpes simplex virus (HSV) has increased in recent years, particularly among women of reproductive age. This places more neonates at risk for severe morbidity and mortality. Treatment recommendations for primary disease in pregnancy are lacking, particularly for those who acquire HSV remote from term.

*Case:* A patient at 17 weeks of gestation carrying dichorionic twins developed primary herpes with subsequent vertical transmission of the virus and significant neonatal morbidity.

*Conclusion:* Data regarding risks and benefits of treatments such as acyclovir and immunoprophylaxis are lacking at a time when the incidence of HSV infection is on the rise. Infect. Dis. Obstet. Gynecol. 5:380–385, 1997. © 1998 Wiley-Liss, Inc.

KEY WORDS
acyclovir, neonatal infection, multiple gestation, pregnancy, chorioretinitis

A recent demographic study of the seroprevalence of type 2 herpes simplex virus (HSV-2) antibody in the United States, conducted between 1988 and 1994, generated the following data: women are significantly more likely to be seropositive than men; during the study period, women in their teens were more likely to be seropositive for HSV-2 than during a prior study conducted between 1976 and 1980; and women of ethnic minority were more frequently seropositive than their white counterparts.[1] As the prevalence of HSV-2 infection among all women of reproductive age has risen significantly, recent efforts have focused on identification of risk factors for neonatal infection. One such study of a large population of women focused on HSV-2 acquisition during pregnancy, and their findings suggested that antiviral chemotherapy may not be necessary if seroconversion occurs prior to the third trimester.[2] The following case report demonstrates that, occasionally, primary HSV-2 infection prior to the third trimester may result in a devastating outcome for the neonate.

### CASE REPORT

The patient was a 26-year-old woman, gravida 3, para 0, with an estimated gestational age of 31.5 weeks at the time of referral to our service for ultrasonographic evaluation of a dichorionic twin gestation. She had had a primary outbreak of genital herpes at 17.5 weeks. At that time, she reported experiencing fever, malaise, and increased vaginal discharge. Her oral temperature was 99.0°F. Her initial pelvic exam revealed no cervical lesions, but there were a significant number of white blood cells noted on the wet-mount culture (vaginal fluid analysis) obtained at that time. There were also several white blood cells noted on urinalysis. She was treated for suspected urinary tract infection with 500 mg of amoxicillin three times a day.

She returned two days later, and a repeat pelvic exam revealed numerous ulcerations on her cervix

*Correspondence to: Dr. Gary M. Joffe, Department of OB-GYN, 5400 Gibson Blvd., S.E., Albuquerque, NM 87108.

Received 24 November 1997
Accepted 3 February 1998

consistent with herpes simplex infection. A culture of these lesions tested positive for herpes. The cultures were performed in the following manner: specimens were vortexed in M5 transport media; centrifugally inoculated onto MRC-5 cells in shell vials; incubated at 35–37°C; and inspected for cytopathic effect daily for five days. The presence of herpes simplex virus (HSV) was confirmed in vials that showed cytopathic effect by HSV-specific fluorescent antibody. The patient was not treated with antiviral chemotherapy at this time. Although the patient's history did not suggest risky behavior, such as intravenous drug abuse or contact with multiple sexual partners, the severity of her infection suggested increased risk for underlying immunosuppression, either on the basis of pregnancy or infection. Testing for human immunodeficiency virus was offered to the patient, but she declined. Her weight gain during pregnancy was appropriate, and her absolute lymphocyte count was 1.5 thousand/ml (normal range, 1.0–4.0 thousand/ml) at the time of evaluation, suggesting lesser likelihood for underlying human immunodeficiency virus infection.

The patient returned one week later reporting significant spontaneous resolution of her symptoms. Serial speculum examinations of the cervix were performed every one to two weeks following initial presentation and revealed the persistence of cervical lesions for a total of 52 days. There was also one episode of labial infection during this period. Three ultrasonographic examinations were performed subsequent to the patient's outbreak of herpes, with the only significant finding being suspicion of lagging abdominal circumference in twin A at 30 weeks of gestation, which prompted referral to our service for further fetal evaluation.

Ultrasonographic examination at 31.5 weeks revealed a dichorionic twin gestation. Twin B, a 1,503-g male, had enlargement of the cerebral atria with drooping choroid plexus. This was not noted on prior examination, but was seen to be present at the 30-week examination upon review of the ultrasound films (Figures 1–4). The range of values obtained for the diameter of the cerebral atria was 10.0–11.8 mm. The lateral ventricular width to hemispheric width ratio and third and fourth ventricles were all within normal limits. The cisterna magna was mildly enlarged, with a measurement of 10.0 mm. Coronal imaging demonstrated normal appearance of the corpus callosum. The remainder of the anatomical survey was normal with the exception of the fetal spleen, with an anteroposterior diameter of 13.5 mm and transverse diameter of 32.5 mm. Twin A, a 1,474-g female, was in a vertex position that precluded examination of the fetal cranium and contents by transabdominal ultrasonographic imaging. The remainder of her anatomical survey was within normal limits. After conferring with the patient's primary caretakers, we placed the patient on a treatment regimen of 200 mg of oral acyclovir five times per day for the remainder of the pregnancy for suspected fetal herpes infection. The remainder of the pregnancy included preterm labor, which was treated with bedrest and oral tocolytic agents. Serial ultrasonographic examinations of twin B demonstrated increasing cerebral ventriculomegaly and progressive cerebellar hypoplasia (Figures 5, 6).

At 35.5 weeks, the patient began active labor and quickly progressed to complete cervical dilation. The patient's perineum and distal vagina were free of visible lesions when she arrived in labor for delivery. Total duration of ruptured membranes surrounding twin A prior to delivery was 4.5 hours. A fetal scalp electrode was placed at the time of rupture of membranes. Vacuum-assisted delivery of twin A was performed for fetal bradycardia, with delivery of an 1,853-g female with an Apgar score of 9 at one minute and 9 at five minutes. Artificial rupture of membranes surrounding twin B was then performed. Umbilical cord prolapse with prolonged fetal bradycardia occurred, and so primary lower uterine segment cesarean delivery was performed, with delivery of an 1,828-g male infant with an Apgar score of 7 at one minute and 8 at 5 minutes. The patient's postoperative recovery was uncomplicated.

Cranial ultrasonography of twin B shortly after birth demonstrated marked dilation of the lateral ventricles and fluid within the ambient cisterns (Figure 7). Figure 8 demonstrates cranial findings consistent with lissencephaly from computed tomography. Ophthalmologic examination obtained on day three of the neonate's life revealed severe bilateral chorioretinitis. Initial eye, oral, and rectal cultures for HSV were negative, and so intravenous acyclovir was discontinued after consultation with

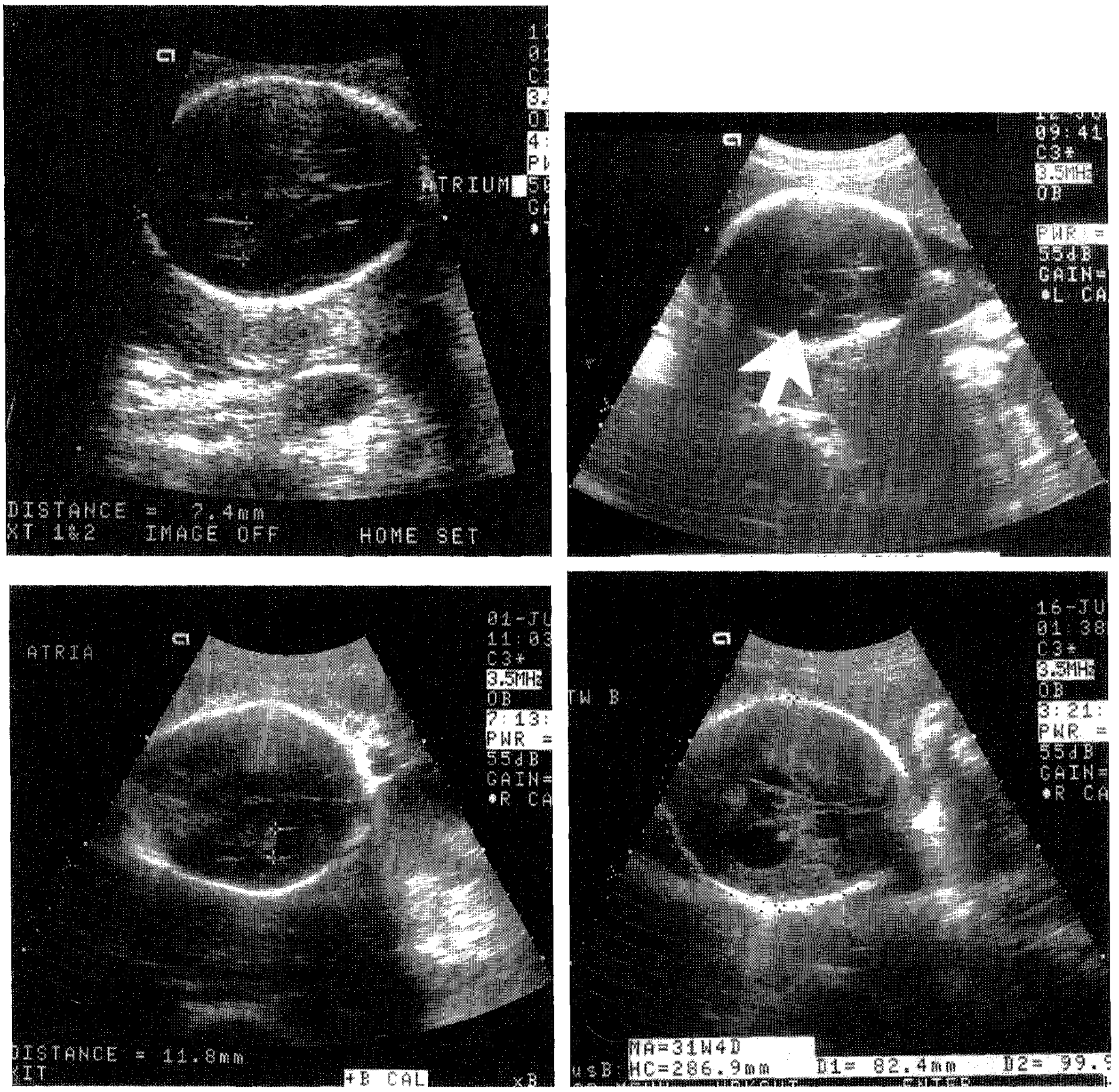

Fig. 1, 2, 3, 4. Figure 1 demonstrates normal dimension of cerebral atria of male fetus at 22 weeks of gestation, 5 weeks after diagnosis of maternal herpes infection. Figures 2, 3, and 4 illustrate the progression of abnormal enlargement of the cerebral atria at 30, 32.5, and 34.5 weeks of gestation, respectively.

pediatric infectious disease specialists. Cerebral spinal fluid was obtained from the male twin on the first day of life, but there was not enough sample available to process for viral culture. A gram stain of cerebrospinal fluid was negative for organisms. Toxoplasma IgM and urine cytomegalovirus were negative. Evaluation of rapid plasma reagin was nonreactive, and the mother was immune to rubella. One month later, however, the male neonate developed a fever and eye and skin lesions suggestive of HSV. Cultures of cerebrospinal fluid and the eye were obtained, and HSV was isolated. Type 1 HSV and type 2 IgG antibody titers were positive at 3.26 and 3.21, respectively; Type 1 HSV and type 2 IgM antibody titers were negative at 0.72 and 0.23, respectively.

The female twin was also noted to have diffuse low density in the cortex and subcortex consistent with diffuse edema and encephalomalacia on cranial computed tomography in the immediate neonatal period, but of lesser severity than her twin (Figure 9). She demonstrated no chorioretinitis at birth or on subsequent examinations. However, HSV was demonstrated by use of polymerase chain reac-

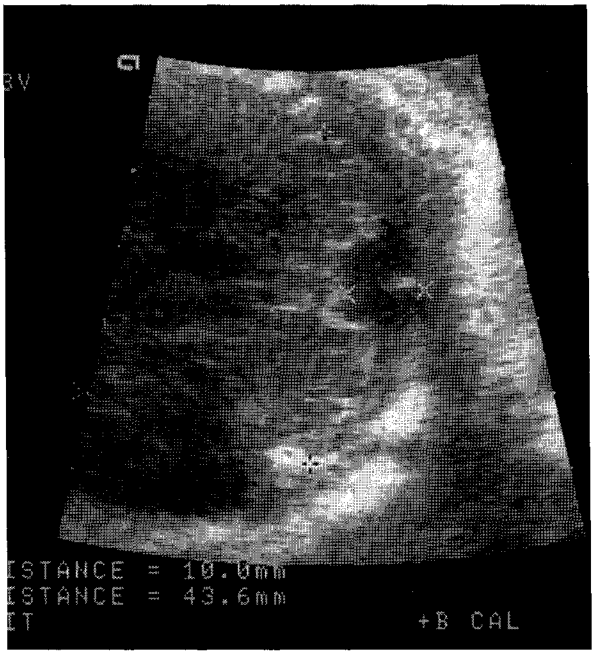

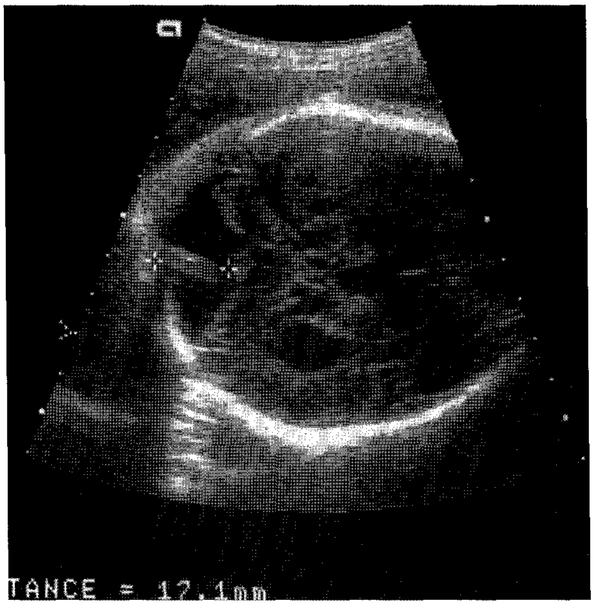

Fig. 5, 6. Figure 5 is a measurement of the cisterna magna of the male fetus at 32.5 weeks, which is at the upper limits of normal for gestational age. Figure 6 demonstrates that at 34.5 weeks there was significant enlargement of the cisterna magna and marked cerebellar atrophy.

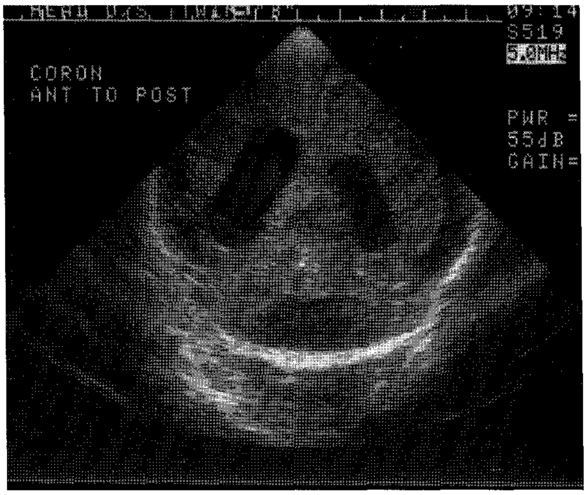

Fig. 7. Figure 7 represents the first cranial ultrasonogram of the male neonate and shows dilation of the lateral ventricles and fluid collections in the ambient cisterns.

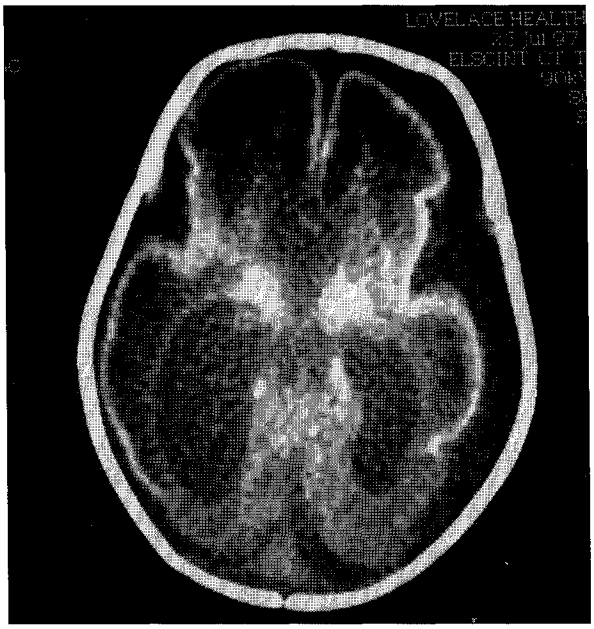

Fig. 8. Cranial computed tomography demonstrating almost complete absence of gyri (lissencephaly) and calcifications in the basal ganglia, along with dilation of the ventricles.

tion testing of her cerebrospinal fluid obtained one month after birth.

### DISCUSSION

This case probably represents primary herpes infection with vertical transmission in the second trimester of pregnancy. Although serologic studies were not obtained at the time of the patient's outbreak, established clinical criteria that suggest primary disease were present. These included the presence of fever and malaise for more than 48 hours and the persistence of genital lesions for greater than 16 days.[3] The male fetus developed progressive severe ventriculomegaly and cerebellar hypoplasia during the late second and early third

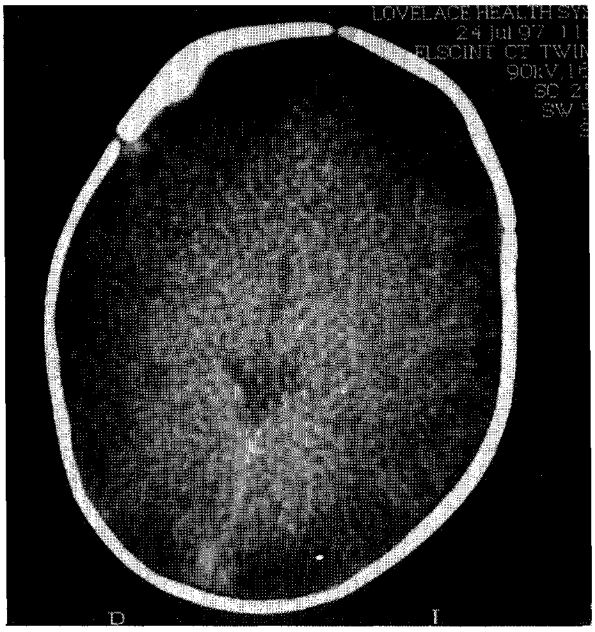

Fig. 9. Cranial computed tomography of the female neonate on the first day of life. Although there were no frank calcifications or ventriculomegaly noted, there is decreased density seen throughout most of the cortex and subcortical white matter, consistent with encephalomalacia or edema.

trimester after documentation of normal intracranial anatomy at 21 weeks. This suggests acute second-trimester insult. These findings in combination with neonatal chorioretinitis are virtually pathognomonic for in utero herpes infection. It must be acknowledged, however, that in the absence of positive neonatal cultures, the pre- and postnatal sonographic and computed tomography findings of long-standing and severe disease could be attributed to another viral type. Likewise, one might argue that the systemic herpes outbreak noted in both twins one month after birth represents postnatal acquisition of virus or horizontal spread between the neonates after twin A had rupture of membranes and placement of fetal scalp electrode. This may be the case for the female infant (twin A), but the male infant was delivered by cesarean within minutes of rupture of membranes, had no fetal scalp electrode applied, and already demonstrated evidence of long-standing in utero infection. Infectious etiology for the severe manifestations noted in the male infant also include chorioamnionitis in membranes that presumably lacked contact with the internal os of the cervix and were therefore less likely to acquire ascending infection from the lower genital tract.

If this case does in fact represent vertical second-trimester transmission, it is disturbing given recent literature suggesting low risk for neonatal morbidity with primary disease remote from term. In perhaps the largest study to date of individuals at risk for acquisition of herpes during pregnancy, only 94 women had documented seroconversion.[2] Furthermore, of the 34 symptomatic women, only 22 became infected during the first or second trimester. In addition, some of these patients received acyclovir. It was clear from this study that patients with third-trimester seroconversion were much more likely to manifest neonatal infection than those infected earlier in pregnancy. It is more difficult, however, to derive the risk for neonatal infection on the basis of only 22 symptomatic patients with first- or second-trimester seroconversion, some of whom may have received acyclovir.

The present case is consistent with a recent case study that suggests that the placenta and/or membranes may be easily traversed by the HSV family of viruses, leaving little evidence for placental infection on histologic examination.[4]

In terms of the obstetric management of this case, several questions may be posed: 1) Why wasn't acyclovir prescribed at the time of initial diagnosis of herpes infection? 2) Were HSV cultures obtained near the time of delivery? 3) Why was cesarean delivery not performed at the onset of labor? and 4) Why was a fetal scalp electrode used with known history of herpes cervicitis?

No randomized clinical trials exist from which to generate recommendations for administration of acyclovir for prevention of vertical transmission. Indeed, it may be tempting to suggest that early and aggressive treatment of the primary maternal herpes would obviate the fetal consequences. This issue is controversial, however, as some authors have suggested that the there may be a delay in the humoral response to HSV after acyclovir therapy.[5] Serial surveillance with weekly cervical cultures for herpes virus has been abandoned due to ineffectiveness in preventing neonatal infection. This policy was adopted by the Infectious Disease Society for Obstetrics and Gynecology, who supports the statement that "in the absence of documented lesions or prodrome, vaginal delivery may be attempted regardless of a history of genital herpes."[5] Examination of the patient in labor should include visualization of the external genitalia, vagina, and cervix, and cesarean delivery should be offered when prodrome or visible lesions are present. There is firm evidence to suggest that the use of fetal scalp electrodes should be avoided in patients with recent seroconversion to an HSV positive state.[6] Even this issue is debated, however, with some authors advocating this form of intrapartum fetal monitoring in the absence of genital lesions.[5]

In summary, this case probably represents second-trimester vertical transmission of herpes simplex virus with devastating outcomes for the neonates. The number of patients with documented primary outbreak of HSV in the second trimester is quite small, and there are currently no large randomized trials of acyclovir in pregnancy to decrease the risk of vertical transmission during the first two trimesters. Therefore, treatment recommendations are lacking. We would suggest that there is a large registry in existence that does give patients and their physicians the option to treat primary outbreaks of HSV in pregnancy in order to attempt to reduce risk of vertical transmission.[7] Without randomized clinical trials, however, the effectiveness and risks of this treatment in prevention of fetal and neonatal disease will remain unknown.

### REFERENCES

1. Fleming DT, McQuillan GM, Johnson RE, et al.: Herpes simplex virus type 2 in the United States, 1976 to 1994. New Engl J Med 337:1105–1111, 1997.
2. Brown ZA, Selke S, Zeh J, et al.: The acquisition of herpes simplex virus during pregnancy. New Engl J Med 337:509–515, 1997.
3. Brown ZA, Vontver LA, Benedetti J, et al.: Effects on infants of a first episode of genital herpes during pregnancy. New Engl J Med 317:1246–1251, 1987.
4. Peng J, Krause PJ, Kresch M: Neonatal herpes simplex virus infection after cesarean section with intact amniotic membranes. J Perinatol 16:397–399, 1996.
5. Cook CR, Gall SA: Herpes in pregnancy. Infect Dis Obstet Gynecol 1:298–304, 1994.
6. Brown ZA, Benedetti J, Ashley R, et al.: Neonatal herpes simplex virus infection in relation to asymptomatic maternal infection at the time of labor. New Engl J Med 324: 1247–1252, 1991.
7. Eldridge R, Andrews E, Tilson H: Pregnancy outcomes following systemic prenatal acyclovir exposure-June 1, 1984–June 30, 1993. MMWR 42:806–809, 1993.

## References

[PDF_00346] Brown Z. A., Benedetti J., Ashley R., Burchett S., Selke S., Berry S., Vontver L. A., Corey L. (1991). Neonatal herpes simplex virus infection in relation to asymptomatic maternal infection at the time of labor.. N Engl J Med.

[PDF_00335] Brown Z. A., Selke S., Zeh J., Kopelman J., Maslow A., Ashley R. L., Watts D. H., Berry S., Herd M., Corey L. (1997). The acquisition of herpes simplex virus during pregnancy.. N Engl J Med.

[PDF_00338] Brown Z. A., Vontver L. A., Benedetti J., Critchlow C. W., Sells C. J., Berry S., Corey L. (1987). Effects on infants of a first episode of genital herpes during pregnancy.. N Engl J Med.

[PDF_00350] Centers for Disease Control and Prevention (CDC) (1993). Pregnancy outcomes following systemic prenatal acyclovir exposure--June 1, 1984-June 30, 1993.. MMWR Morb Mortal Wkly Rep.

[PDF_00344] Cook C. R., Gall S. A. (1994). Herpes in pregnancy.. Infect Dis Obstet Gynecol.

[PDF_00332] Fleming D. T., McQuillan G. M., Johnson R. E., Nahmias A. J., Aral S. O., Lee F. K., St Louis M. E. (1997). Herpes simplex virus type 2 in the United States, 1976 to 1994.. N Engl J Med.

[PDF_00341] Peng J., Krause P. J., Kresch M. (1996). Neonatal herpes simplex virus infection after cesarean section with intact amniotic membranes.. J Perinatol.

